# Investigating proactive aggression in patients with borderline personality disorder and major depressive disorder using a modified version of the Taylor aggression paradigm

**DOI:** 10.3389/fpsyg.2024.1439924

**Published:** 2024-12-13

**Authors:** Sara Boccadoro, Philippa Hüpen, Adrian Raine, Ute Habel, Lisa Wagels

**Affiliations:** ^1^Department of Psychiatry, Psychotherapy and Psychosomatics, Faculty of Medicine, RWTH Aachen University, Aachen, Germany; ^2^JARA-BRAIN Institute Brain Structure and Function, INM-10, Institute of Neuroscience and Medicine, Jülich Research Centre, Jülich, Germany; ^3^Department of Criminology, Psychiatry and Psychology, University of Pennsylvania, Philadelphia, PA, United States

**Keywords:** proactive aggression, major depressive disorder, borderline personality disorder, psychophysiology, skin conductance

## Abstract

**Introduction:**

Inappropriate reactive (provoked) aggression is common in various psychiatric disorders, including Borderline Personality Disorder (BPD) and, to a lesser extent, Major Depressive Disorder (MDD). Less is known about proactive (unprovoked) aggression in these patients, with mixed findings in the literature. Drawing from the current evidence, we expect higher trait aggression in both patient groups and higher behavioral proactive aggression and physiological arousal in patients with BPD compared to both MDD and healthy participants (HC).

**Methods:**

We investigated behavioral and psychophysiological correlates of proactive aggression in 23 patients with MDD, 20 with BPD, and 21 HC using a proactive version of the Taylor Aggression Paradigm (pTAP). The pTAP consists of reaction time games in which only the participant can interfere with the ostensible opponent’s performance by modifying the blurriness of the opponent’s screen. The levels of blurriness chosen by participants reflect their proactive aggression. We collected self-report measures of aggression and other personality traits. We further adopted a transdiagnostic approach by clustering participants based on proactive aggression characteristics.

**Results:**

Both patient groups reported higher trait aggression than HC but not higher aggression in the task nor differences in the associated physiological arousal. Trial-by-trial mixed model analyses revealed that the group characterized by higher proactive aggression traits behaved more aggressively after losing, suggesting a role of frustration or sensitivity to loss.

**Discussion:**

Our study confirms that patients with MDD and BPD report higher aggression than HC despite the absence of observable behavioral and psychophysiological differences and highlights the ubiquity of proactive aggression characteristics across diagnoses.

## 1 Introduction

Exaggerated or inappropriate aggression is commonly reported in a variety of psychiatric disorders, including Borderline Personality Disorder (BPD) and Major Depressive Disorder (MDD; Blair, 2018; Nelson and Trainor, 2007; Newhill et al., 2009). Two subtypes of aggression are frequently distinguished: a reactive form, which occurs in response to a provocation, and a proactive form, which is instrumental and unprovoked (Baron and Richardson, 1994; Dodge et al., 1997; Dodge, 1991). In adult psychiatric patients, research frequently documents noticeable problems in reactive aggression in patients with different psychiatric disorders, including BPD and MDD. The literature on MDD indicates a high prevalence of anger and aggressive outbursts in patients with MDD (Liu and Cole, 2021; de Bles et al., 2019) and also more externally directed aggression and irritability compared to healthy controls (HC; Fritz et al., 2020). Patients with BPD also seem to be at risk for reactive aggression (Blair, 2018) and externalizing, aggressive behavior (Sansone and Sansone, 2012), as well as higher self-assessed levels of trait anger and aggression than HC (Cackowski et al., 2017).

Given the high correlation between self-reported reactive and proactive aggression (Polman et al., 2007), prior research suggests that proneness to one type of aggression implies a tendency also toward the other type (Verona and Bresin, 2015). While this overlap has led to criticisms of the distinction between proactive and reactive aggression (Bushman and Anderson, 2001), several studies support the distinction and demonstrate its clinical relevance (Brugman et al., 2017; Fung et al., 2009; Polman et al., 2007; Card and Little, 2006; Ferguson and Dyck, 2012; Chester, 2024). The distinction is beneficial for capturing the different underlying supporting motivations, understanding the distinct developmental trajectories of these forms of aggression, and tailoring interventions accordingly. Rather than excluding the existence of both forms, the overlap suggests that they often co-occur. As such, patients with MDD and BPD might also be inclined to proactive aggressive behaviors. However, little research exists on proactive aggression in these disorders.

The relationship between proactive aggression and depressive symptoms remains unclear. Some evidence suggests a positive link between depressive symptoms and proactive aggression in children from primary (Rieffe et al., 2016) and secondary schools (Roland, 2002), as well as adolescents (Yang et al., 2023). Other studies query these findings, documenting that proactive aggression is not significantly related to negative affect and internalizing problems such as depressive symptoms and suicidal behavior (Fite et al., 2009; Fite et al., 2014). These differences in results may be explained by the fact that the latter studies were conducted with child psychiatric inpatients and exclusively male adolescents, respectively. In contrast, the former studies included school children and adolescents of both genders.

Depression is often linked with egocentrism (Baron and Hanna, 1990; Erle et al., 2019), which implies a reduced willingness or ability to consider other’s perspectives, and a reduction in empathy and perspective-taking. Reduced perspective-taking abilities can in turn lead to selfishness (Raine and Uh, 2019), which is related to personality traits known as the Dark Triad, which is also associated with various forms of aggression (Deutchman and Sullivan, 2018; Jones and Neria, 2015). Selfishness itself has been associated with proactive aggressive behavior (Boccadoro et al., 2021). Thus, depression might be related to proactive aggression through egocentrism and selfishness. This hypothesis requires further investigation.

Similarly, inconsistent evidence emerges from studies in patients with BPD. Both reactive and proactive aggression measured via questionnaires correlate with BPD symptomatology (Thomson and Beauchaine, 2019; Gardner et al., 2012; Ostrov and Houston, 2008). Patients with BPD often show behavioral characteristics that may be considered as proactive aggression, such as manipulating others to achieve a goal (Zanarini et al., 2007). Indeed, proactive and reactive relational aggression were associated with BPD symptoms in a sample of young adults (Ostrov and Houston, 2008).Yet, proactive aggression may be significantly correlated with BPD traits only when considering raw aggression scores on the proactive aggression scale of the Reactive-Proactive Aggression Questionnaire (RPQ), but not when using residualized scores of proactive aggression, obtained by regressing reactive aggression scores on proactive aggression scores, as a measure of “pure” proactive aggression (Gardner et al., 2012). Similarly, Thomson and Beauchaine (2019) reported a positive correlation between BPD symptoms and proactive aggression scores, but this effect disappeared when accounting for reactive aggression.

Patients with BPD often report high levels of depressive symptoms (Beatson and Rao, 2013), which may contribute to proactive aggressive behavior through impaired perspective-taking and increased selfishness, as previously discussed. Similarly to patients with depression, patients with BPD have difficulties with perspective-taking (Colle et al., 2019). However, whether patients with BPD exhibit higher levels of selfishness traits remains to be investigated.

Furthermore, gender differences may play an important role in moderating the association between proactive aggression and depression and borderline symptomatology. A study on intimate partner violence perpetrators diagnosed with antisocial personality disorder indicated that men with comorbid BPD engaged more in reactive rather than proactive violence (Ross and Babcock, 2009). Considering the gender differences in both depression and BPD, with men showing more externalizing symptoms, such as aggressiveness, and women showing more internalizing symptoms, gender might influence proactive aggression in both patients’ groups (Qian et al., 2022; Martin et al., 2013).

In summary, the current literature is inconsistent, with studies reporting an association between proactive aggression and MDD and BPD and other studies reporting no such association, especially when accounting for reactive aggression. More research is needed, as studies on proactive aggression in MDD and BPD is scarce and contradictory. Importantly, the reported studies were performed only including self-reported questionnaires or parents’ reports, which capture trait, but not state, aggression and can be susceptible to bias. On the contrary, laboratory aggression paradigms allow measuring state (in-the-moment) aggression.

One of the most frequently used aggression paradigms is the Taylor Aggression Paradigm (TAP; Taylor, 1967), designed to capture state reactive aggression. In the TAP, participants compete in several reaction time games against an ostensible opponent, who provokes them by administering a punishment [e.g., aversive noise, heat stimuli, or monetary deductions; see Weidler et al. (2019) and Krämer et al. (2007)]. Reactive aggression is measured by recording the levels of punishment selected by the participants for their opponent in response to the provocation. Studies using the TAP and similar tasks showed that patients with BPD react more aggressively than HC to provocations (Kogan-Goloborodko et al., 2016; McCloskey et al., 2009; New et al., 2009). In addition, Kogan-Goloborodko and colleagues reported higher overall aggression in patients with BPD compared to HC. However, another study using the Social Threat Aggression Paradigm reported that patients with BPD did not show higher state aggression than HC (Bertsch et al., 2022).

Proactive aggression has been difficult to measure via laboratory paradigms. This limits our knowledge of the physiological and psychological correlates of proactive aggressive behaviors. Recently, some groups have developed and validated proactive aggression paradigms by modifying and testing the TAP in healthy volunteers (Zhu et al., 2019; Boccadoro et al., 2021). In both studies, participants competed against an ostensible opponent in a reaction time task and could impair the opponent’s performance by either auditory (Zhu et al., 2019) or visual (Boccadoro et al., 2021) interference, without ever being provoked by the opponent. The interference levels selected by the participants serve as a measure of proactive aggression, as they are instrumentally used to damage the opponent and increase participants’ chances of winning and getting a reward.

Boccadoro and colleagues further reported that state proactive aggression was associated with a reduced skin conductance. The finding is in line with studies on trait proactive aggression (Armstrong et al., 2019), in psychopathy and conduct disorder (Lorber, 2004) and on state proactive aggression in children (Moore et al., 2018; Hubbard et al., 2010). However, other studies did not identify this association (Fanti, 2018; Hubbard et al., 2002; Banny et al., 2014; Scarpa et al., 2010). Skin conductance is a sensitive measure of autonomic physiological arousal, and although non-specific, it is a correlate of aggression (Critchley and Nagai, 2013; Christopoulos et al., 2019; Bechara and Damasio, 2005; Lorber, 2004).

Skin conductance has been used to investigate autonomic dysfunction in MDD (Schumann et al., 2017; Schneider et al., 2012; Iacono et al., 1983; Kim et al., 2019; Smith et al., 2020; Sarchiapone et al., 2018) and patients with BPD (Aleknaviciute et al., 2016; Banny et al., 2014; Barnow et al., 2012; Baschnagel et al., 2013; Herpertz et al., 2000; Hüpen et al., 2020). Most of these studies document reduced skin conductance in MDD but increased skin conductance in BPD. Yet, findings are mixed in both patient groups, as some studies reported increased skin conductance in patients with MDD (Schneider et al., 2012; Schumann et al., 2017), and reduced skin conductance in patients with BPD (Herpertz et al., 2000), or no effects in patients with BPD (Baschnagel et al., 2013). Most studies on MDD used cross-sectional designs and included both male and female patients, with sample sizes ranging from 11 to 30 patients. While the sample sizes and study designs are similar, discrepancies in findings appear to be related to variations in the conditions under which skin conductance was measured, such as during rest, stress induction, or emotion-processing tasks. Nevertheless, the majority of the literature, as reviewed by Sarchiapone et al. (2018), supports the finding of hypoactivation in patients with MDD.

Similarly, most studies in BPD adopted cross-sectional designs, included sample sizes of 16 to 33 patients (mostly women), and measured skin conductance under different conditions. Discrepancies in findings emerge in studies recording skin conductance during exposure to emotional stimuli (Baschnagel et al., 2013; Herpertz et al., 2000; Barnow et al., 2012), while studies measuring skin conductance during stress, risk-taking or at rest consistently indicated hyperarousal in women and girls with BPD (Banny et al., 2014; Aleknaviciute et al., 2016; Hüpen et al., 2020). This suggests that different paradigms detect different arousal patterns in patients with BPD. Gender differences could also contribute to these discrepancies. These inconsistent findings highlight the need for testing whether and how peculiar patterns of skin conductance are linked to aggression in MDD and BPD, and how these patterns differ from HC.

Given that it is unclear how patients behave in a competitive context measuring aggression, the present study investigates trait aggression and state proactive aggression (aggression in the paradigm) in patients with MDD and with BPD as well as HC while recording skin conductance response (SCR). We expect higher general trait aggression in both patient groups than in HC. We expect higher state proactive aggression in patients with BPD compared to patients with MDD and HC, accompanied by higher SCR, indicating hyperarousal. In addition, we aim to explore whether SCR patterns during the task can differentiate between the three groups and whether patients with MDD would behave differently from HC in the paradigm. Lastly, we adopt an exploratory transdiagnostic approach by clustering participants based on characteristics related to proactive aggression. This allows us to identify potential risk factors for proactive aggression, regardless of the diagnoses. This approach aligns with growing evidence that moving beyond traditional diagnostic systems better captures the complexity and dimensionality of clinical reality (Dalgleish et al., 2020).

## 2 Materials and methods

### 2.1 Participants

Twenty-three participants with MDD (mean age = 35.17, *SD* = 12.61, 17 women), 22 participants with BPD (mean age = 29.36, *SD* = 8.61, 19 women) and 25 HC (mean age = 26.83, *SD* = 9.28, 19 women) were recruited for the current study. Four of the 22 patients with BPD had comorbidity with MDD, but here are included in the BPD group given that BPD is their primary diagnosis. Participants of the patients’ groups were recruited through the Department of Psychiatry, Psychotherapy and Psychosomatics of the RWTH Aachen University Hospital. HC were recruited via public flyers, social networks, mailing lists, and contact lists with volunteers from previous studies at the University Hospital RWTH Aachen.

All participants met the following inclusion criteria: aged between 18 and 50 years, sufficient German language skills, no current substance use or addiction and no neurological diseases. An additional inclusion criterion for the clinical groups was the presence of MDD and of BPD as the primary psychiatric diagnosis, respectively. The exclusion criterion for HC was any history of psychiatric disorders. Before coming to the testing session, all participants were asked to complete an online screening. Eligible participants for the three groups were invited for a diagnostic session with the Structured Clinical Interview for DSM-5 (SCID-5; First, 2015).

Two participants (HC) were excluded from the study because they did not complete the paradigm, four participants (2 HC and 2 BPD) due to a high number of missing responses (> 10 trials) in the proactive aggression task and four additional participants (3 HC and 1 MDD) from SCR analyses due to problems during acquisition of the SCR data. Hence, the final sample for the behavioral aggression analyses included 23 patients with MDD (mean age = 35.17, *SD* = 12.61, 6 males), 20 patients with BPD (mean age = 29.40, *SD* = 8.76, 3 males) and 21 HC (mean age = 27.65, *SD* = 9.70, 4 males). The final sample for the SCR analyses included 22 patients with MDD (mean age = 35.05, *SD* = 12.89, 6 males), 20 patients with BPD (mean age = 29.40, *SD* = 8.76, 3 males) and 18 HC (mean age = 26.00, *SD* = 7.80, 4 males).

In those from the final sample (behavioral aggression analyses), 17 patients with MDD and 17 patients with BPD were on psychotropic medications. Most patients with MDD and with BPD had one or more current comorbid diagnoses. Information on medications and the most common comorbid diagnosis in our sample is included in Supplementary Table 1.

The study was approved by the Ethics Committee of the Medical Faculty of the University Hospital RWTH Aachen University in June 2021 (Reference number: EK20-215). All participants provided written informed consent according to the Declaration of Helsinki and were paid 35 € as financial reimbursement for their participation.

### 2.2 Paradigm description: proactive Taylor aggression paradigm (pTAP)

During the test session, participants performed three behavioral tasks (a risk-taking task and a trust game were performed after the aggression task and are not examined in this manuscript), and their skin conductance response (SCR) was recorded during the entire test period. In the first task, the pTAP, participants engaged in a competitive reaction time task against an ostensible opponent, matched on gender. Due to the ongoing SARS-CoV-2 pandemic, participants could not meet their ostensible opponent in person. Therefore, before the beginning of the task, the experimenter made a fake phone call to coordinate with an ostensible colleague. Participants were told that the colleague was simultaneously measuring the opponent in the same task to make the competition believable. Participants were also told that they have been pre-assigned to a specific “role,” which allows only them, and not the opponent, to choose blurriness levels for the screen of the opponent during the game, thus interfering with the opponent’s performance. In total, the task consisted of 40 trials. Participants knew that each trial was worth two euros and that five trials out of 40 would be randomly extracted at the end of the paradigm to determine the total reward for the participants, with a maximum reward of 10 euros. Therefore, a higher winning rate in the task would increase the chance of getting a higher reward.

**Figure 1:**
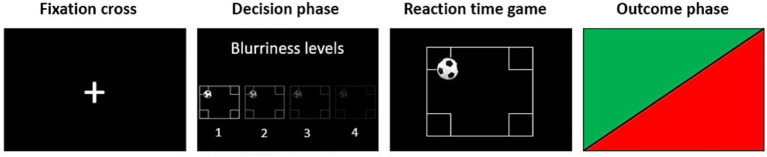
Visual description of one trial of the proactive Taylor Aggression Paradigm (pTAP). During the *fixation cross* phase, participants were asked to look at a fixation cross on the screen. During the *decision phase*, participants had 5 s to select the level of interference for the opponent on a level from 1 (no blurriness) to 4 (maximum blurriness). In the *reaction time game*, participants had to press a button as fast as possible when they saw a ball entering any of the target areas at the corners of the field. During the *outcome phase*, participants saw whether they won or lost the reaction time game in that trial by seeing a flash of green or red light, respectively. Participants played a total of 40 trials.

A visual description of the paradigm is presented in Figure 1. In each trial, participants were first presented with a fixation cross, then they were asked to choose the level of screen blurriness for the opponent within 5 s (decision phase). The levels available ranged from 1 (normal screen) to 4 (maximum blurriness) and there was no limit to the number of times each level could be selected. Examples of how the screen of the opponent would look like in the four different blurriness conditions were presented before the beginning of the task in some instruction trials and always available during the decision phase. After the decision phase, participants were presented with another fixation cross which preceded the reaction time task, in which participants were instructed to press a button as fast as possible when a ball bounced against any of the four corners at the borders of the screen. The game was followed by the outcome phase, in which a flash of green or red light, respectively, indicated that participants won or lost the game (outcome phase).

The paradigm was programmed and presented using the Presentation® software of neurobehavioral systems^1^. Unknown to the participants, the task was preprogrammed to associate each level of blurriness with a certain probability of winning the trial. Level one corresponded to a 30% chance of winning the trial, level two to a 50% chance, level three to a 70% chance, and level four to a 90% chance. The whole procedure, including explanation of the task, four instruction trials and the paradigm, lasted approximately 15 min.

Since the groups of participants were pre-determined by their diagnosis, and the chances of winning and losing were determined by participants’ choices, resulting in different percentages of loss and won outcomes across participants, our study follows a quasi-experimental design.

### 2.3 Personality traits and depression self-assessment

After the paradigm, participants were asked to complete a series of questionnaires to assess several neuropsychological variables, such as trait aggression, and selfishness. Self-reported trait aggression was assessed by the RPQ (Raine et al., 2006) and the Buss Perry Aggression Questionnaire (BPAQ; Buss and Perry, 1992), while selfishness was assessed by the Selfishness Questionnaire (SQ; Raine and Uh, 2019). Depressive symptoms were assessed by the Beck Depression Inventory-II (BDI-II; Steer and Clark, 1997, German version: Hautzinger et al., 2010). Further questionnaires were administered, including the Sensitivity to Punishment/Sensitivity to Reward Questionnaire (SPSRQ; Torrubia et al., 2001), the Barratt Impulsiveness Scale (BIS-11; Patton et al., 1995; Stanford et al., 2009), the Domain-Specific Risk-Taking Scale (DOSPERT-G; Johnson et al., 2004), and the Positive and Negative Affect Schedule (PANAS; Krohne et al., 2014). In addition, participants’ strategy, and belief in the cover story for the pTAP were assessed with a self-developed questionnaire. All questionnaire data were collected via SoSci Survey®^2^. For the objectives of the current study, we incorporated only data related to the RPQ, BPAQ, SQ, and BDI-II into analyses, as well as belief in the cover story.

### 2.4 Psychophysiological data acquisition

SCR was recorded with the Brain Vision Recorder (Brain Products GmbH, Gilching, Germany)^3^. Measures were taken by using two grounded flat silver/silver chloride (Ag-AgCl) electrodes (10 mm diameter), prepared with a 0.5% saline paste in a neutral base (Med Associates TD-246), placed on the middle phalanges of the index and middle finger of participants’ non-dominant hand and instructing the participants to rest their hand on the desk in the most comfortable position, avoiding any movements. Data were recorded at 5,000 Hz and a direct current excitation voltage of 0.5 V. The recording of SCR was synchronized with the pTAP task sequence using triggers sent by the Presentation® software (see text foot note 1). SCR was recorded continuously during the paradigm.

### 2.5 SCR data preprocessing and analysis

SCR data were preprocessed with BrainVision Analyzer by sampling the data to 20 Hz and visually inspecting the data to adjust for movement artifacts. Data were then exported and analyzed using the Ledalab toolbox (V.3.4.9) based on standardized procedures as recommended, which includes smoothing using the Gauss-method and a window width of 16 samples and data filtering applying a low-pass Butterworth filter with a 2 Hz cutoff (Benedek and Kaernbach, 2010). The data analysis was performed using a continuous decomposition analysis (CDA) to decompose the SCR data into continuous phasic and tonic activity. The CDA method follows four steps: (a) estimation of a parameter describing tonic activity, (b) non-negative deconvolution of phasic SCR data resulting in a driver function and a non-negative remainder, (c) segmentation of the driver and the remainder to identify single impulses by peak detection, and (d) reconstruction of the SCR data.

As our aim was to investigate SCR underlying the time course of decision-making in proactive aggression, we extracted the time integral of the phasic driver over the entire decision phase of the pTAP and used it as dependent variable for the trial-by-trial mixed model analysis. The phasic driver time integral represents the cumulative phasic activity within a specified response window (Benedek and Kaernbach, 2010). The response window ranged from 1 to 5 s after condition presentation (start of the decision phase). A minimum amplitude criterion of 0.05 μS was used for peak detection.

### 2.6 Statistical analyses

Descriptive statistics were analyzed using SPSS 25.0 software. To calculate mean levels of state proactive aggression we averaged the blurriness levels chosen in the pTAP across all 40 trials. To check whether the data were normally distributed the Shapiro–Wilk test was used. For non- normally distributed data, non-parametric tests were used for the analyses. Additionally, given the high correlation between trait proactive and reactive aggression reported in the literature (Card and Little, 2006; Raine et al., 2006; Polman et al., 2007; Fite et al., 2009), we tested the bivariate correlation between the two subscales of the RPQ (proactive and reactive subscales) using Spearman’s rho correlation. The correlation was significant (*r_s_* = 0.705, *p* < 0.001). Therefore, to obtain a measure of “pure” trait proactive aggression independent of reactive aggression, we computed the residuals of trait proactive aggression (Raine et al., 2006), by regressing trait reactive aggression on trait proactive aggression scores and saving Pearson standardized residuals (mean = 0, SD = 1). We will refer to this “pure” trait proactive aggression as *residual proactive aggression* in this manuscript.

A Chi-square test of independence was performed to examine whether the groups differed regarding gender and belief in the cover story. The Kruskal-Wallis test (H-test) was used to check whether HC, patients with MDD and patients with BPD differed on age, trait proactive aggression in the RPQ, residual proactive aggression in the RPQ, selfishness in the SQ (data in the SQ were not normally distributed in the HC group), the different subscales of the BPAQ, depression scores in the BDI-II, and SCR during the decision phase. Pairwise comparisons using Dunn’s test and Bonferroni correction were conducted as *post hoc* test. One-way ANOVA was conducted to test whether HC, patients with MDD and patients with BPD differed for state proactive aggression in the task, and for trait reactive aggression in the RPQ. Tukey’s Honestly Significant Difference (HSD) Test for multiple comparisons was used as *post hoc* test.

Pearson correlations were conducted to test the relationships between mean levels of state proactive aggression and self-reported measures of selfishness in the SQ and of hostility in the BPAQ. Spearman’s rho correlations were conducted to test the relationships between mean levels of state proactive aggression and self-reported measures of trait proactive aggression in the proactive aggression subscale of the RPQ, *residual proactive aggression* in the RPQ, physical aggression in the BPAQ, and depression score in the BDI-II. Bonferroni correction for multiple comparisons was applied. The same correlations described in this section were further computed for each group separately applying Bonferroni correction.

Participants’ descriptions of their strategy in the game were categorized based on their motivations for selecting blurriness levels. These categories were then analyzed qualitatively to determine whether state aggression was driven by proactive aggression-related motivations or by other motivations. An exploratory one-way ANOVA was run to compare the three categories based on their state proactive aggression. A chi-square test was run to test if HC, MDD and BPD differed regarding their motivations.

### 2.7 Robust linear mixed-effects model analyses

To investigate whether the three groups (HC, patients with MDD and patients with BPD) differ in state proactive aggression, and in their SCR during decision making related to proactive aggression in the task, two separate linear mixed- effects trial-by-trial models were fitted in RStudio (RStudio Team, 2020), using the *lme4* package (Bates et al., 2015). Then, the normality of distribution of the residuals for each model was tested using the Shapiro–Wilk test. Since the assumption of normality of residuals distribution was violated (*W* = 0.993, *p* < 0.001 and *W* = 0.719, *p* < 0.001, respectively), two robust linear mixed- effects models were run (Model 1 and Model 2 respectively). Only the results of these robust models will be discussed in this manuscript. Corresponding *p* values for statistical tests were obtained using the *lmerTest* package (Kuznetsova et al., 2017) with the significance level set at an alpha level of 0.05. Effect sizes for significant effects were calculated using the *effectsize* package to extract the standardized coefficients (*β*). State proactive aggression levels in the task were treated as a continuous predictor using single data points for each trial instead of computing the average as opposed to the basic statistical analyses. In SCR models, “non-responders” were included in the analyses, since small variations of SCR or a lack of SCR to stimuli may be the product of biological processes that underlie individual differences (Marin et al., 2020).

Model 1 estimated state proactive aggression (*Aggrchoice*) as a function of the fixed effects *Group* (HC, MDD, and BPD), O*utcome* (win vs. loss in the previous trial), *Gender* (men vs. women), and *Trial.z* (the trial variable was z-transformed for comparability of parameter estimates in the model). Furthermore, to test whether game outcome in the previous trial would differentially influence state proactive aggression in the different groups, the interaction term Group * Outcome was included. The model included random slopes for trials and a random intercept for participants (*Subject*).

Model 1 ← rlmer(Aggrchoice ~ Group + Outcome + Gender + Trial.z + Group*Outcome + (1 + Trial.z|Subject), data = pTAP, method = “DASvar”).

Model 2 estimated SCR as a function of the fixed effects *Aggrchoice* (from 1 to 4), *Group* (HC, MDD, and BPD), O*utcome* (win vs. loss), and *Gender* (men vs. women). As in model 1, state proactive aggression levels were treated as single data points for each trial (*Aggrchoice*). To test whether state proactive aggression would differentially influence SCR in the different groups, the interaction term Group * Aggrchoice was included. The interaction term *Group* * *Outcome* was added to test whether game outcome in the previous trial would differentially influence SCR in the different groups. The model included a random intercept for participants and random slopes for trials.

Model 2 ← rlmer(SCR ~ Aggrchoice + Group + Outcome + Gender + Trial.z + Group*Aggrchoice + Group*Outcome + (1 + Trial.z|Subject), data = pTAP, method = “DASvar”).

Additionally, since our sample included a large majority of women compared to men, we performed sub-analyses including only women (17 per group). Therefore, we ran two additional models for state proactive aggression and SCR (Model S1 and Model S2 respectively), which are identical to Model 1 and 2, excluding the fixed effect *Gender*.

### 2.8 Cluster analyses

A hierarchical clustering with Ward’s method using squared Euclidean distance was performed using SPSS 25.0 to cluster our sample based on *residual proactive aggression* in the RPQ, and Z-scores of self-reported levels of selfishness in the SQ. These scores were chosen to cluster our sample based on characteristics related to proactive aggression, including selfishness, which was found to be related to state proactive aggression in a previous study using the pTAP in healthy volunteers (Boccadoro et al., 2021). Mean levels of state proactive aggression in the task, SCR in the decision phase, hostility, anger, physical and verbal aggression in the BPAQ, trait reactive aggression in the RPQ, and depression scores were compared between the two identified transdiagnostic groups by using independent samples t-test for normally distributed data and Mann–Whitney U test for non-normal data. Moreover, Chi-square tests were used to test whether the two transdiagnostic groups differed in their distribution regarding group membership based on the diagnostic categories (HC, patients with MDD and patients with BPD), gender and belief in the cover story (yes, no). The same correlations described in the group analyses were further computed for each identified transdiagnostic group separately applying Bonferroni correction.

Lastly, we ran two additional robust linear-mixed effects models (Model 3 and Model 4) for state proactive aggression and SCR respectively, which are identical to models 1 and 2, but include the transdiagnostic groups (*Cluster*) obtained from the cluster analyses instead of the groups for a transdiagnostic comparison.

Model 3 ← rlmer(Aggrchoice ~ Cluster + Outcome + Gender + Trial.z + Cluster*Outcome + (1 + Trial.z|Subject), data = pTAP, method = “DASvar”).

Model 4 < − rlmer(SCR ~ Aggrchoice + Cluster + Outcome + Gender + Trial.z + Cluster*Aggrchoice + Cluster*Outcome + (1 + Trial.z|Subject), data = pTAP, method = “DASvar”).

## 3 Results

### 3.1 Group differences on proactive aggression and SCR

Information on descriptive statistics is included in Table 1. The RPQ, BPAQ, and SQ in the current study showed good internal consistency (RPQ: Cronbach’s *α* = 0.876; BPAQ: Cronbach’s *α* = 0.851; SQ: Cronbach’s α = 0.820).

**Table 1:** Demographic and descriptive statistics of the final sample included in the study.

	HC (*n* = 21)	MDD (*n* = 23)	BPD (*n* = 20)	Stats.	*p*	*p* (pairwise)
Age (years)	27.43 ± 9.51	35.17 ± 12.61	29.40 ± 8.76	*H* (2) = 4.31	0.12	
Gender (women)	17	17	17	*X*^2^ (2, *N* = 64) = 0.84	0.66	
Handedness (right)	19	21	18	*X*^2^ (2, *N* = 64) = 0.02	0.99	
Belief in cover story (yes)^a^	8	8	14	*X*^2^ (1, *N* = 62) = 5.52	0.06	
State proactive aggression (pTAP, choices from 1 to 4)	2.41 ± 0.81	2.07 ± 0.78	2.18 ± 0.54	*F* (2, 61) = 1.27	0.29	
BDI-II	4.76 ± 4.61	26.74 ± 10.54	30.90 ± 14.21	*H* (2) = 34.38	< 0.001***	MDD > HC*** BPD > HC***
RPQpro	0.57 ± 1.03	0.87 ± 1.22	2.05 ± 2.35	*H* (2) = 8.01	< 0.05*	BPD > HC*
RPQre	4.96 ± 3.17	6.78 ± 3.62	10.20 ± 5.38	*F* (2, 61) = 8.46	< 0.001***	BPD > HC** BPD > MDD*
residual RPQpro	−0.01 ± 0.75	−0.13 ± 0.77	0.16 ± 1.39	*H* (2) = 0.57	0.75	
Hostility (BPAQ)	12.19 ± 3.96	17.43 ± 4.60	22.70 ± 6.45	*H* (2) = 26.53	< 0.001***	MDD > HC** BPD > HC***
Anger (BPAQ)	14.48 ± 3.63	15.00 ± 4.91	19.55 ± 5.84	*H* (2) = 8.78	< 0.05*	BPD > HC* BPD > MDD*
Physical aggression (BPAQ)	13.33 ± 2.54	16.91 ± 4.35	17.05 ± 5.90	*H* (2) = 9.19	< 0.05*	MDD > HC*
Verbal aggression (BPAQ)	12.71 ± 3.78	11.96 ± 3.27	13.05 ± 4.51	*H* (2) = 1.12	0.57	
SQ	12.52 ± 8.01	16.00 ± 8.26	15.95 ± 6.34	*H* (2) = 3.53	0.17	
SCR^b^ (decision phase)	0.26 ± 0.18	0.25 ± 0.25	0.26 ± 0.26	*H* (2) = 0.50	0.78	

HC, healthy controls; MDD, patients with major depressive disorder; BPD, patients with borderline personality disorder; pTAP, proactive Taylor Aggression Paradigm; RPQpro, proactive aggression subscale of the RPQ; RPQre, reactive aggression subscale of the RPQ; residual RPQpro, residuals of the proactive aggression subscale of the RPQ; SCR, skin conductance response.

^a Data regarding Belief in cover story were missing for two patients with MDD.^

^b Data regarding SCR were missing for 3 HC and 1 patient with MDD.^

*p < 0.05; **p < 0.01; ***p < 0.001.

There were no significant differences between HC, patients with MDD, and patients with BPD regarding age, gender, belief in the cover story, and handedness (Table 1).

**Figure 2:**
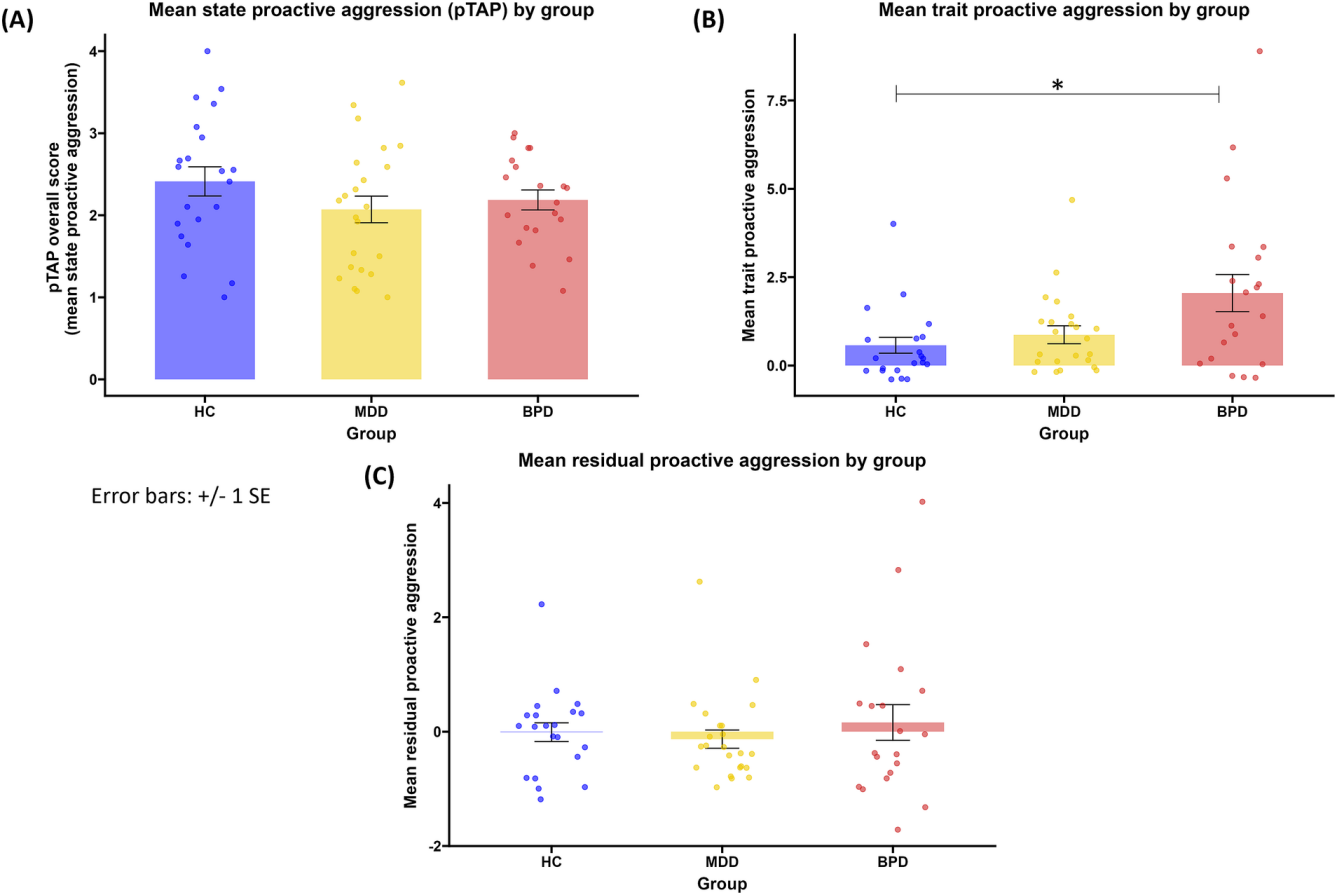
**(A)** mean state proactive aggression by group; **(B)** mean trait proactive aggression (scores in the proactive aggression subscale of the RPQ) by group; **(C)** mean residual proactive aggression (standardized residuals of the proactive aggression subscale of the RPQ) by group. HC, healthy controls; MDD, major depressive disorder, BPD: borderline personality disorder. *p < 0.05.

Analyzing the subscales of the RPQ showed group differences. The groups differed in trait proactive aggression [*H*(2) = 8.006, *p* = 0.018, Figure 2]. Patients with BPD reported significantly higher trait proactive aggression in the RPQ compared to HC (*z* = 2.785, *p* = 0.016), while no significant difference emerged between other groups (BPD vs. MDD: *z* = 1.886, *p* = 0.178, MDD vs. HC: *z* = 0.972, *p* = 0.993). No significant difference between groups emerged when residual proactive aggression was compared [*H*(2) = 0.574, *p* = 0.751, Figure 2]. Trait reactive aggression differed in groups as well [*F*(2, 61) = 8.461, *p* < 0.001]. The *post hoc* test revealed that the trait reactive aggression was significantly higher in patients with BPD compared to HC (*M* = 5.248, *SE* = 1.289, *p* < 0.001) and patients with MDD (*M =* 3.330, *SE* = 1.262, *p* = 0.028). There was no significant difference between the patients with MDD and HC (*M* = 1.917, *SE* = 1.246, *p* = 0.280).

Analyzing the subscales of the BPAQ (hostility, anger, physical and verbal aggression) revealed group differences in three out of four subscales [hostility: *H*(2) = 26.528, *p <* 0.001; anger: *H*(2) = 8.781, *p* = 0.012; physical aggression: *H*(2) = 9.193, *p* = 0.010], but not in verbal aggression [*H*(2) = 1.119, *p* = 0.572]. Both patients with BPD and patients with MDD reported higher hostility in the BPAQ than HC (MDD vs. HC: *z* = 3.069, *p* = 0.006; BPD vs. HC: *z* = 5.120, *p* < 0.001), while no significant difference emerged between patients’ groups (BPD vs. MDD: *z* = 2.202, *p* = = 0.083). Patients with BPD reported higher anger in the BPAQ than both HC (*z* = 2.575, *p* = 0.030) and patients with MDD (*z* = 2.597, *p* = 0.028), while HC and patients with MDD did not differ (*z* = 0.035, *p* = 1.000). Patients with MDD reported higher physical aggression than HC (*z* = 2.850, *p* = 0.013), while no differences emerged between other groups (MDD vs. BPD: *z* = 0.429, *p* = 1.000, BPD vs. HC: *z* = 2.333, *p* = 0.059).There was not a statistically significant difference between groups regarding state proactive aggression [*F*(2.61) = 1.267, *p* = 0.289, Figure 2] and its accompanied SCR patterns(*H*(2) = 0.497, *p* = 0.780). There was no statistically significant difference between groups regarding self-reported levels of selfishness [*H*(2) = 3.529, *p* = 0.171]. Groups differed in the BDI-II scores [*H*(2) = 34.382, *p* < 0.001]. The *post hoc* test showed that patient groups reported a significantly higher depression compared to HC (BPD: *z* = 5.365, *p* < 0.001; MDD: *z* = 4.752, *p* < 0.001), while patients with BPD and patients with MDD did not differ in depression scores (*z* = 0.792, *p* = 1.000).

Results of the correlations between state proactive aggression and the questionnaires scores in either all participants or in each group separately are included in the Supplementary Table 2. The only significant finding after correcting for multiple comparisons was a positive correlation between state and trait proactive aggression, but not residual proactive aggression, in the RPQ in patients with MDD.

A total of 18 participants (7 HC, 8 MDD, 3 BPD) described a strategy for their decisions regarding blurriness levels.

Seven participants [2 HC, 4 MDD, 1 BPD; mean state proactive aggression = 3.00, *SD* = 0.52, 95% CI (2.52, 3.48)] reported motivations aligned with proactive aggression, such as choosing higher blurriness levels to win or make the task more difficult for the opponent. Six participants [3 HC, 2 MDD, 1 BPD; mean state proactive aggression = 2.24, *SD* = 0.68, 95% CI (1.53, 2.95)] indicated mixed motivations, combining elements of reactive aggression and fairness. For example, they adjusted blurriness levels to increase their chances of winning after losing, then decreased the levels when winning more frequently, or experimented with different levels to determine the point at which they could win, before later selecting lower levels to ensure a fair match. Five participants [2 HC, 2 MDD, 1 BPD; mean state proactive aggression = 1.78, *SD* = 0.65, 95% CI (0.98, 2.58)] were solely motivated by fairness. An exploratory one-way ANOVA showed a significant difference between the three different strategies [*F*(2) = 6.186, *p* = 0.011]. Tukey post-hoc test indicates a significant difference in state proactive aggression between participants reporting motivations for proactive aggression and those driven by fairness (*M* = 1.220, *SE* = 0.357, *p* = 0.010), but not those reporting mixed motivations (*M* = 0.761, *SE* = 0.339, *p* = 0.096). Participants reporting mixed motivations and fair motivations did not differ in state proactive aggression (*M* = 0.459, *SE* = 0.369, *p* = 0.447). A chi-square test to compare HC, patients with MDD and patients with BPD based on their self-reported strategy did not show any significant difference [*X*^2^ (4, *N* = 18) = 0.884, *p* = 0.927].

In summary, the analyses revealed no significant differences among the groups in state proactive aggression, physiological arousal during the decision phase, or trait selfishness. However, significant differences were found in depression symptomatology and trait aggression. Participants also reported different motivations for their behavior.

### 3.2 Associations between proactive aggression and SCR in the groups

The results of models 1 and 2 are listed in Supplementary Table 3. Model 1 did not reveal any significant effect. Model 2 revealed a significant main effect of *Trial.z* [*t* = −6.71, *p* < 0.001; *β* = −0.12, 95% CI = (−0.15, −0.08); Supplementary Figure 1], indicating that SCR habituated during the paradigm. Moreover, the model revealed a significant interaction effect between *Group* and *Aggrchoice* (*t* = 2.08, *p* < 0.05). *Post hoc* tests for this interaction were not significant (HC vs. MDD: *z* = −0.099, *p* = 0.995; HC – BPD: *z* = −2.077, *p* = 0.095; MDD – BPD: *z* = −2.063, *p* = 0.098), indicating no significant differences in the arousal patterns associated with proactive aggression in the three groups. A plot of the interaction effect is available in the Supplementary Figure 2.

The results of the sub-analyses in women are listed in Supplementary Table 4. Model S1 showed a significant interaction effect between *Group* and *Outcome* [*t* = 2.92, *p* < 0.01; *β* = 0.27, 95% CI = (−0.09, 0.45)]. *Post hoc* tests for this interaction showed that women with MDD behaved more aggressively following losing compared to winning (won vs. loss: *z* = −3.611, *p* < 0.001). No significant difference between won and loss outcome emerged in HCs (*z* = 0.580, *p* = 0.562) and in BPD (*z* = −1.416, *p* = 0.157), and there were no significant differences between groups in either won or loss outcome conditions. *Post hoc* test results are available in Supplementary Table 6. Model S2 revealed only a significant main effect of Trial.z [*t* = −5.68, *p* < 0.001; *β* = −0.13, 95% CI = (−0.17, −0.08)], indicating that SCR habituated during the paradigm.

Our analyses revealed that only in the female subsample, patients with MDD exhibit more aggressive behavior following losses than wins in the game, a pattern not observed in the whole sample. No associations with specific arousal patterns were found.

### 3.3 Differences between the identified transdiagnostic groups

**Figure 3:**
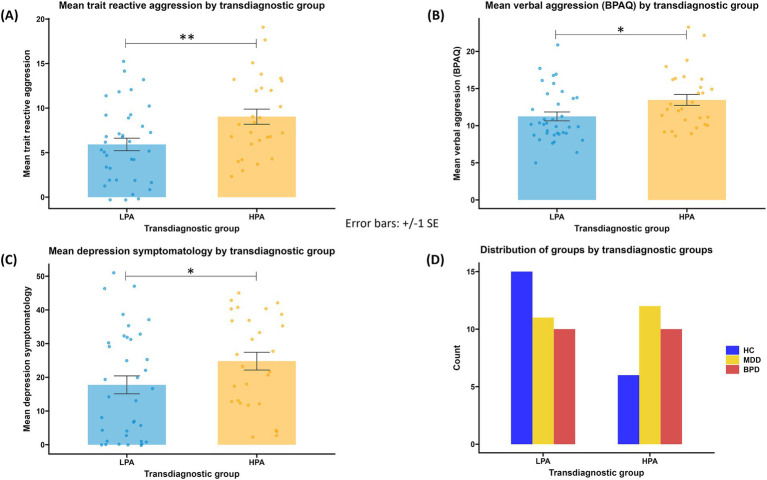
**(A)** mean trait reactive aggression (scores in the reactive aggression subscale of the RPQ) by transdiagnostic group; **(B)** mean verbal aggression (scores in the verbal aggression subscale of the BPAQ) by transdiagnostic group; **(C)** mean depression symptomatology (depression scores in the BDI-II) by transdiagnostic group; **(D)** composition of the LPA and HPA groups regarding the diagnostic groups (HC, healthy controls; MDD, major depressive disorder, BPD: borderline personality disorder). LPA: low proactive aggression group; HPA, high proactive aggression group. *p < 0.05; **p < 0.01.

The cluster analysis revealed two transdiagnostic groups (Supplementary Figure 3), one low in characteristics related to proactive aggression (LPA, *n* = 36) and one high in characteristics related to proactive aggression (HPA, *n* = 28). LPA and HPA groups did not differ in their composition regarding the diagnostic group [HC, MDD, or BPD; *X*^2^ (1, *n* = 64) = 2.947, *p* = 0.229; Figure 3]. The two transdiagnostic groups did not differ regarding the belief in the cover story [*X*^2^ (1, *n* = 62) = 0.625, *p* = 0.429]. Information on descriptive statistics is included in Supplementary Table 4. The HPA group reported higher verbal aggression in the BPAQ (*U* = 319.5, *p* = 0.012), higher trait reactive aggression as reported in the RPQ [*t*(62) = 2.845, *p* = 0.006], and higher depression scores (*U* = 356.5, *p* = 0.046) than the LPA group (Figure 3). The two transdiagnostic groups did not differ in self-reported hostility (*U* = 411, *p* = 0.207), anger (*U* = 379.5, *p* = 0.091) and physical aggression in the BPAQ (*U* = 415, *p* = 0.225). No correlation between state proactive aggression and any questionnaire within each transdiagnostic group was significant (Supplementary Table 2).

**Figure 4:**
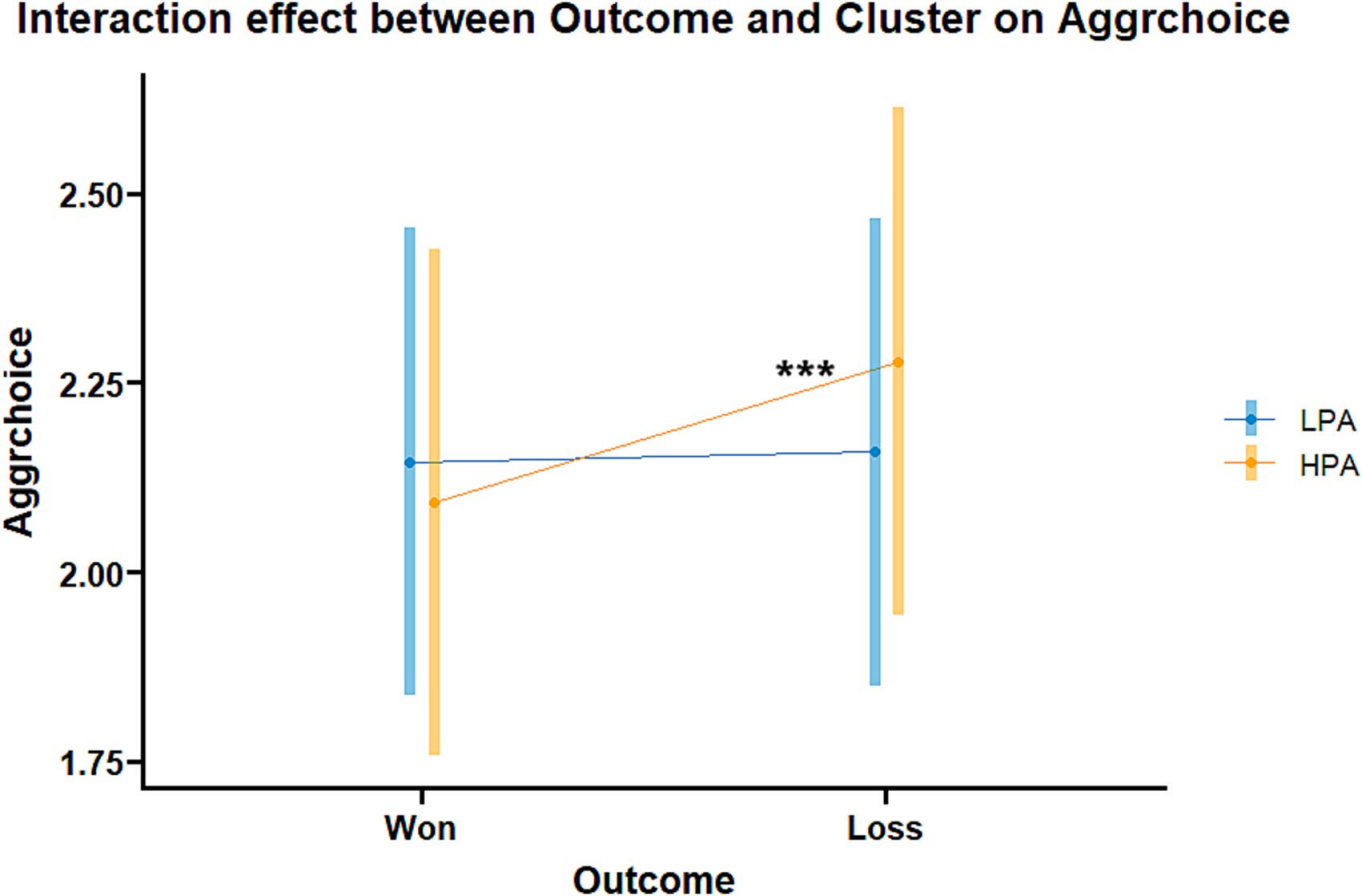
Interaction effect between *Outcome* and *Cluster* in the aggression choice model in the transdiagnostic groups (Model 3). Aggrchoice: state proactive aggression; LPA, low proactive aggression group; HPA, high proactive aggression group. ***p < 0.001.

The results of models 3 and 4 are listed in Table 2. Model 3, testing aggressive choices in the pTAP, revealed a significant interaction effect between *Cluster* and *Outcome* [*t* = 2.55, *p* < 0.05; *β* = 0.15, 95% CI = (0.04, 0.27); Figure 4]. *Post hoc* tests for this interaction revealed state proactive aggression in the HPA group to be higher after losing compared to winning (*z* = 3.658, *p* < 0.001). No significant difference between won and loss outcome emerged in the LPA group (*z* = −0.283, *p* = 0.777), and no significant differences emerged between the transdiagnostic groups in either won (*z* = 0.263, *p* = 0.793) or loss (*z* = −0.583, *p* = 0.560) outcome conditions. Model 4, testing differences in the SCR, revealed a significant main effect of Trial.z [*t* = −6.81, *p* < 0.001; *β* = −0.12, 95% CI = (−0.16, −0.09)], indicating that SCR habituated during the paradigm.

**Table 2:** Parameter estimates from the analyses testing differences in transdiagnostic groups.

Model 3 (Aggchoice) rlmer [Aggrchoice ~ Cluster + Outcome + Gender + Trial.z + Cluster*Outcome + (1 + Trial.z|Subject)]					
Fixed effects	*b*	SE	*95%* CI	*t*	*p*
Intercept	2.09	0.24	1.61–2.56	8.60	< 0.001***
Cluster (HPA)	−0.05	0.20	−0.45 – 0.35	−0.26	0.793
Outcome (Loss)	0.01	0.04	−0.08 – 0.10	0.28	0.777
Gender (Women)	0.12	0.25	−0.37 – 0.61	0.49	0.627
Trial z	−0.04	0.03	−0.10 – 0.03	−1.13	0.260
Cluster (HPA) × Outcome (Loss)	0.17	0.07	0.04–0.31	2.55	0.011*

Model 4 (SCR) rlmer [SCR ~ Aggrchoice + Cluster + Outcome + Gender + Trial.z + Cluster*Aggrchoice + Cluster*Outcome + (1 + Trial.z|Subject)]					
Fixed effects					
Intercept	0.22	0.04	0.15–0.30	5.82	< 0.001***
Aggrchoice	0.01	0.01	−0.00 – 0.02	1.35	0.177
Cluster (HPA)	−0.01	0.03	−0.08 – 0.06	−0.25	0.805
Outcome (Loss)	−0.01	0.01	−0.03 – 0.01	−0.99	0.320
Gender (Women)	−0.02	0.03	−0.09 – 0.04	−0.67	0.506
Trial z	−0.06	0.01	−0.08 – −0.04	−6.81	< 0.001***
Aggrchoice × Cluster (HPA)	−0.02	0.01	−0.03 – 0.00	−1.72	0.085
Cluster (HPA) × Outcome (Loss)	0.01	0.02	−0.02 – 0.05	0.87	0.386

b, estimate; SE, Standard Error; CI, Confidence Intervals; Aggrchoice, state proactive aggression; Cluster (HPA), High Proactive Aggression group; Outcome, game outcome in the previous trial; Trial.z, z-transformed trials. *p < 0.05; ***p < 0.001.

Our results show that the transdiagnostic group characterized by higher proactive aggression-related traits reports greater levels of trait reactive aggression, verbal aggression, and depression symptomatology compared to the group low in proactive aggression-related traits. Additionally, this high-proactive aggression group exhibits more aggressive behavior following losses in the game compared to when they win.

## 4 Discussion

Investigating proactive aggression and its associated psychophysiological correlates in both patients with MDD and patients with BPD allows for a comparative and transdiagnostic analysis to identify potential shared and distinct mechanisms in the manifestation of proactive aggression. Regarding the distinct mechanisms, our findings indicate that patient groups’ self-reports on general aggression are higher than self-reports by HC. For patients with BPD this also includes trait proactive and reactive aggression specifically. Patients with BPD also estimated higher general and reactive aggression than patients with MDD. In contrast, our results do not indicate any behavioral differences between patient groups and HC in levels of proactive aggression in the task nor in their associated psychophysiological patterns. Patients and HC did not differ in selfishness traits.

Across groups, participants characterized by higher proactive aggression characteristics (HPA) reported higher depression levels than those with lower proactive aggression and showed a selective increase in behavioral aggression following a losing game. This finding suggests that frustration due to losses might be a shared mechanism of aggressive behaviors in people with higher proactive aggression characteristics and depression symptomatology across diagnoses. Nevertheless, aggression following losses was not higher in the HPA group compared to the LPA group.

### 4.1 Distinct mechanisms

Both patients with MDD and patients with BPD frequently report high occurrences of anger and aggression (de Bles et al., 2019; Kogan-Goloborodko et al., 2016; Liu and Cole, 2021; McCloskey et al., 2009; Neukel et al., 2022). In line with previous studies, patients with MDD described themselves as more aggressive than HC. However, patients with MDD did neither report higher trait proactive aggression than HC nor showed higher arousal or aggression in the task. This evidence supports the conclusion that proactive aggression in MDD is not a symptom of the pathological manifestation of this mental disease. However, our sub-analyses in women revealed that women diagnosed with MDD exhibit higher proactive aggression after losing compared to winning. These results hint at a role of frustration in promoting proactive aggression specifically in this group, although a full gender comparison is lacking due to the limited number of men in our sample. Nonetheless, aggression following losing in women with MDD was not higher than that observed in HC.

Interestingly, there was a positive correlation between trait proactive aggression and aggression in the task specific to patients with MDD. It is unclear whether these results reflect a higher awareness in patients with MDD of their own behavioral and personality characteristics, or if patients with MDD tend to behave in ways that align with their self-perception, such as displaying higher proactive aggression when they see themselves as aggressive. This should be explored in future studies.

Patients with BPD reported higher general trait aggression and reactive aggression than HC and even than patients with MDD as previously reported in patients with BPD with or without comorbid MDD (Soloff et al., 2000). Importantly, patients with BPD reported higher proactive aggression than HC. This could be a specific and distinct characteristic of individuals with BPD, although no behavioral and psychophysiological correlates were observed. However, when accounting for reactive aggression, patients with BPD did not report higher proactive aggression than HC, similar to what has been shown in previous studies (Gardner et al., 2012; Thomson and Beauchaine, 2019). This finding might indicate that the higher proactive aggression reported by patients with BPD is strongly influenced by their higher reactive aggression scores, which are reflected in actual higher reactive aggressive behaviors as reported by previous studies (Kogan-Goloborodko et al., 2016; McCloskey et al., 2009; New et al., 2009; Verona and Bresin, 2015). Previous research suggests that proneness to one type of aggression implies a tendency to the other type as well (Verona and Bresin, 2015). In our study, this seems to be specific to patients with BPD.

Despite reporting higher trait aggression, patients with BPD did not show higher aggression in the task, either in general, nor after losing or winning the game, in line with a previous study (Bertsch et al., 2022) A potential explanation for this might be that patients with BPD have a bias in their self-perception. This bias would lead them to perceive themselves as acting more aggressively than they do. One contributing factor may be the disruption in self-image, which is a common feature of BPD. These patients often exhibit more negative self-attributes than HC (Vater et al., 2015). As a result, they may be more likely to interpret their actions as aggressive, even when their behavior does not objectively reflect this.

Alternatively, our task might involve other aspects, including competition, that might overlay proactive aggression, leading to other motivations for participants’ behavior. We attempted to assess participants’ motivations regarding their choices of blurriness levels. The few participants who described their strategies reported different motivations. Some aimed for fairness, while others were driven by proactive aggression, seeking to win at the expense of their opponent. Others exhibited a mix of proactive and reactive aggression, along with fairness motivations, as they reported increasing blurriness after losing and reducing it after winning. This suggests frustration from defeat, a desire to win, but a wish to maintain some fairness. These findings indicate that motivations in the task extend beyond proactive aggression alone. However, as only three patients with BPD described their strategies, no conclusions can be drawn about their specific motivations, warranting further investigation in future studies. In summary, self-reported higher trait aggression, with proneness to both proactive and reactive aggression, seems to be a specific mechanism in patients with BPD, distinguishing them from both HC and patients with MDD, despite not being reflected at the behavioral level in the present task.

### 4.2 Shared mechanisms

We further adopted a transdiagnostic approach to identify two distinct groups based on characteristics related to proactive aggression, specifically residual proactive aggression (which is a measure of proactive aggression independent from reactive aggression), and selfishness scores in the questionnaires, irrespective of diagnostic categories. One group was characterized by higher levels of proactive aggression-related traits (HPA), and another was characterized by lower levels (LPA). Depression, verbal aggression and trait reactive aggression were related to the HPA group, similarly to what was previously observed (Dutton and Karakanta, 2013; Rieffe et al., 2016). This suggests an association between aggression and depression, and further highlights how different types of aggression co-occur, in line with previous findings (Verona and Bresin, 2015). As the two groups did not differ in their composition regarding the diagnosis, our analysis suggests that traits related to proactive aggression are ubiquitous in patients and healthy participants.

The two transdiagnostic groups did not show discernible differences in behavioral aggression nor its accompanied psychophysiological arousal, indicating no specific risk marker for state proactive aggression across diagnosis. However, participants in the HPA group behaved more aggressively following a loss game outcome, similarly to women with MDD. Thus, we observed a heightened reaction to frustration among participants in the HPA group. Instead of proactive aggression, this reaction rather points to a reactive aggressive process as described in the frustration-aggression hypothesis, which was recently reformulated as a “quest for significance” (Kruglanski et al., 2023). In the revised theory, the authors propose that a loss of one’s sense of significance (i.e., personal worth, social worth, dignity) via frustration, rejection, or humiliation can lead to aggression as a means to restore the lost sense of significance when more socially accepted alternatives are less salient or unavailable. Losing the game in our task might thus elicit frustration, resulting in a loss of significance (as loss of competence) in participants in the HPA group, who then react aggressively against the opponent to regain their sense of significance, as no alternative way to restore significance is available. This explanation aligns with the higher trait reactive aggression reported by the participants in the HPA group compared to those in the LPA group. Previous research also supports this interpretation, showing increased aggression when losing in a competitive video game (Breuer et al., 2015).

The increased aggression following a loss outcome might not only be driven by frustration, but also by the wish to reduce the risk of further losses in the subsequent game. The higher depression scores reported by the HPA group compared to the LPA group might support both these explanations. Indeed, previous studies reported higher loss sensitivity and lower frustration tolerance in participants with higher depression symptomatology (Berry et al., 2019; Mahon et al., 2007). This is consistent with our finding of increased aggression following losses in women with MDD. Alternatively, the higher selfishness traits in the HPA group could drive aggressive choices aimed at maximizing the chances of winning the subsequent game and, thus, getting a reward at the opponent’s expense. However, no correlation between selfishness traits and behavioral aggression in this group emerged, suggesting that the frustration-aggression hypothesis and loss sensitivity might better explain this response to losses.

Interestingly, the two transdiagnostic groups did not differ in composition based on the classical diagnostic criteria. This finding suggests that proactive aggression characteristics are ubiquitous across diagnoses, at least those examined in the current study, as well as in healthy individuals. Accumulating evidence over the last 10–15 years has questioned the validity of the classic diagnostic criteria due to the high heterogeneity of mental disorders and shared characteristics across different conditions. The Research Domain Criteria (RDoC) project has encouraged moving beyond the classic diagnostic categories to study psychopathology through dimensional constructs, spanning from normality to abnormality, by integrating multiple *units of analysis*, such as behavioral, physiological, and neurobiological data (Morris et al., 2022; Cuthbert, 2022). Our transdiagnostic approach aligns with RDoC goals by identifying risk factors for proactive aggression across diagnostic boundaries and integrating behavioral, physiological, and self-report data. Our findings support the necessity of moving beyond traditional diagnostic labels in favor of transdiagnostic, dimensional frameworks and contribute to advancing RDoC-oriented research by shedding light on the transdiagnostic risk factors underlying proactive aggression. Our findings suggest that having higher proactive aggression traits and higher depression symptomatology predisposes to higher loss sensitivity and higher frustration due to losses which result in higher aggression in response to frustration.

### 4.3 Recommendations for future research

A review on sex differences in BPD hinted at a possible role of gender in moderating the association between proactive aggression and BPD symptoms since men tend to show more externalizing symptoms, such as aggression, compared to women (Qian et al., 2022). Our analysis including gender in the model did not show any effect of gender. However, our sub-analysis in women showed an effect of loss outcomes which was not identified in the whole sample. The higher proportion of women compared to men included in the study might drive the results and mask potential gender differences, which could be unraveled by future studies including a larger number of men diagnosed with MDD and BPD. For instance, previous research has shown an association between proactive aggression and lower physiological arousal, particularly in healthy men, whereas healthy women tend to exhibit the opposite pattern (Boccadoro et al., 2021). Including more men with BPD and MDD could help clarify whether gender influences these associations in a similar way as previously observed in healthy populations.

Our analysis did not identify any significant differential patterns of associations between SCR and aggression in the groups at the post-hoc level. However, the significant interaction in the model shows a trend in the direction of different SCR patterns (see Supplementary Figure 2). Specifically, patients with BPD seem to show higher physiological arousal with increasing aggression compared to both patients with MDD and HC, whose patterns look similar. Given the clinical relevance of such association, we recommend future studies to replicate these analyses with larger sample sizes to test whether there is indeed a different association between physiological arousal and proactive aggression in patients with BPD.

Lastly, MDD and BPD are mainly characterized by reactive aggression, whereas proactive aggression is more common in violent offenders and individuals with antisocial personality disorder. Given the strong correlation between reactive and proactive aggression (Polman et al., 2007) and the dimensional nature of proactive aggression, it remains important to explore this form of aggression in patients with BPD and MDD as well. However, future studies could include groups associated with higher proactive aggression for better comparisons. Our study design would be well-suited to examine state proactive aggression and the related physiological arousal patterns in individuals with antisocial personality disorder or in violent offenders.

### 4.4 Limitations

It is essential to consider some limitations when interpreting the results of the current study. The results revealed that in participants included in the HPA group, and in the subsample of women with MDD, loss outcomes elicited higher aggression in the task, indicating a frustration element or heightened sensitivity to loss. Hence, the behavior in the task may be a mixture of proactive and reactive aggression, which are highly intercorrelated (Card and Little, 2006; Raine et al., 2006; Polman et al., 2007; Fite et al., 2009). As aggressive behaviors can stem from various motivations (Runions et al., 2018), our task focuses on the primary motivation of proactive aggression (i.e., reward-seeking, goal-directed) without excluding other motivations.

Additionally, the proactive aggression scores in our study were right-skewed, as reported in other studies (Fite et al., 2024), indicating that most participants reported very low levels of proactive aggression, often scoring 0 or near 0. Previous research suggests that the RPQ is more accurate in assessing proactive and reactive aggression in male adolescents and young adults than in adult women (Tobar et al., 2020), although another study found the RPQ to be reliable in adult men (Brugman et al., 2017). Since our sample consists mostly of adult women, it is possible that the RPQ has limitations in capturing proactive aggression within this sample. As a result, the skewed distribution of proactive aggression scores in our study may reduce the effect sizes obtained from our analyses, potentially masking stronger true effects.

Most patients were taking psychotropic medication, which could potentially impact the behavior and psychophysiology of patients. However, discontinuing medication for research purposes may raise ethical concerns, and the use of psychotropic medication reflects real-world conditions for these patient groups.

Lastly, we acknowledge the modest sample size of our study, which limits the statistical power of our analysis and may account for the non-significant trends observed. We conducted a post-hoc power analysis using G*power (Faul et al., 2009), approximating our robust linear mixed-effects models to a linear multiple regression. For model 3 (64 participants, 5 predictors), the analysis indicates a power of 11% for a small effect size (0.02), 61% for a medium effect (0.15), and 96% for a large effect (0.35). Although power is limited for medium effects in our study, our calculation was based on an approximation. The robust linear-mixed models used in our analysis can account for both between- and within-subject variability, as well as for the error terms of data points coming from the same source (repeated measures of the same participants) thus increasing power compared to simpler models (Aarts et al., 2015; Algermissen and Mehler, 2018). Future studies with larger sample sizes could validate our findings and further explore the SCR patterns in patients with BPD.

## 5 Conclusion

Our study supports previous findings reporting that patients with BPD and patients with MDD tend to describe themselves as more aggressive than HC. Yet, we showed that behavioral proactive aggression and its associated psychophysiological correlates are comparable in patients and HC. Our findings emphasize the need for a transdiagnostic approach to studying aggression, as it is a common behavior across various psychiatric disorders and even among healthy individuals. People with the same diagnosis can vary considerably in aggression, or individuals with different diagnoses can show similar aggression traits or behaviors. This variability would be overlooked if we only focused on the specific diagnosis. While our two identified transdiagnostic groups did not differ in state proactive aggression nor the associated physiological arousal patterns, preventing the identification of specific markers related to proactive aggression, they showed a different behavioral response to losses in the game. The effect size for this finding was 0.15, indicating a small effect (Nieminen, 2022), consistent with studies utilizing the TAP (West et al., 2021). This small effect size warrants caution in interpreting the results, highlighting the need for replication in a larger sample. These findings suggest that motivations driving proactive aggression behaviors (goal-directed) may intersect with those motivating reactive aggression, such as frustration, particularly in individuals with heightened proactive aggression tendencies and higher depression symptomatology, supporting prior research findings.

## Data Availability

The datasets presented in this study can be found in online repositories. The names of the repository/repositories and accession number(s) can be found at: https://github.com/sboccadoro/MDD-BPD_pTAP.

## Footnotes

^1^ www.neurobs.com

^2^ https://www.soscisurvey.de/

^3^ https://www.brainproducts.com/index.php
