# Supplementary material for: Investigating proactive aggression in patients with borderline personality disorder and major depressive disorder using a modified version of the Taylor aggression paradigm
Source: Front Psychol. 2024 Dec 13;15:1439924. doi: 10.3389/fpsyg.2024.1439924 (PMC11672799; doi:10.3389/fpsyg.2024.1439924)
Supplement: Supplementary file 1 [file Table_1.docx]

Supplementary Material

## Supplementary Tables

**Supplementary Table 1.**List of medications and the most common comorbid diagnosis in our sample.

|  |  | **MDD (*n* = 23)** | **BPD (*n* = 20)** |
| --- | --- | --- | --- |
| Psychotropic medication |  | 17 | 17 |
| SSRIs |  | 8 | 5 |
| SSNRIs |  | 6 | 3 |
| Antipsychotics |  | 5 | 6 |
| Atypical  antidepressants |  | 3 | 4 |
| Stimulants |  | 1 |  |
| Anticonvulsants |  |  | 2 |
| Tricyclic  antidepressants |  |  | 1 |
| Opioid  antagonists |  |  | 1 |
| Benzodiazepines |  |  | 1 |
| Phenothiazines |  |  | 1 |
| Comorbidity |  |  |  |
| Panic disorder |  | 10 |  |
| ADHD |  | 9 | 2 |
| Agoraphobia |  | 8 |  |
| PTSD |  | 5 | 11 |
| GAD |  | 5 | 6 |
| SAD |  | 4 |  |
| Eating disorders |  |  | 8 |
| Obsessive  thoughts |  |  | 3 |
| MDD |  |  | 4 |
| Depressive  symptoms |  |  | 11 |

MDD, major depressive disorder; BPD, borderline personality disorder, SSRIs, selective serotonin reuptake inhibitors; SSNRIs, selective serotonin and norepinephrine reuptake inhibitors; ADHD, attention deficit hyperactivity disorder; PTSD, posttraumatic stress disorder; GAD, generalized anxiety disorder; SAD, social anxiety disorder.

**Supplementary Table 2.**Correlations between state proactive aggression and questionnaires scores in the whole sample, in the three different groups, and in the two identified transdiagnostic groups (LPA = Low Proactive Aggression, HPA = High Proactive Aggression).

| **State proactive aggression** | **Hostility**  **(BPAQ)** | | **Physical aggression (BPAQ)** | | **RPQpro** | | **residual RPQpro** | | **SQ** | | **BDI-II** | |
| --- | --- | --- | --- | --- | --- | --- | --- | --- | --- | --- | --- | --- |
| ***Whole sample*** | ***r*** | ***p*** | ***r_s_*** | ***p*** | ***r_s_*** | ***p*** | ***r_s_*** | ***p*** | ***r*** | ***p*** | ***r_s_*** | ***p*** |
| **All** | -.10*^c^* | .44 | -.19 | .13 | .10 | .43 | .11 | .37 | .05 | .69 | -.13 | .30 |
| **HC** | -.15 | .51 | -.27 | .23 | -.13 | .57 | .05 | .82 | .25***^a^*** | .27 | .04 | .87 |
| **MDD** | .08 | .73 | .21 | .34 | .57 | .004** | .29 | .18 | .05 | .84 | -.14*^b^* | .52 |
| **BPD** | -.10 | .67 | -.50 | .02*^a^* | -.09 | .69 | .01 | .98 | -.12 | .60 | .13*^b^* | .58 |
| ***Transdiagnostic groups*** | |  |  |  |  |  |  |  |  |  |  |  |
| **LPA** | -.05 | .76 | -.17 | .38 | .04 | .84 | -.04*^b^* | .82 | .08 | .64 | .01 | .96 |
| **HPA** | -.23*^c^* | .24 | -.22 | .25 | -.18 | .36 | .28 | .14 | .13 | .52 | -.38 | .049*^a^* |

*^a^* Does not survive Bonferroni correction for multiple comparisons with alpha = .008

*^b^* Pearson correlation was used instead of Spearman’s rho correlation

residual RPQpro: residuals of proactive aggression in the RPQ (residual proactive aggression)

*^c^* Spearman’s rho correlation was used instead of Pearson correlation

**Supplementary Table 3.**Parameter estimates from the linear mixed-effects model analyses for aggression choices (Model 1) and for SCR (Model 2).

| **Model 1 (Aggrchoice)**  *rlmer (Aggrchoice ~ Group + Outcome + Gender + Trial.z + Group*Outcome +*  *(1 + Trial.z\|Subject))* | | | | | |
| --- | --- | --- | --- | --- | --- |
| **Fixed effects:** | ***b*** | ***SE*** | ***95% CI*** | ***t*** | ***p*** |
| Intercept | 2.28 | 0.27 | 1.75 – 2.80 | 8.50 | <.001*** |
| Group (MDD) | -0.37 | 0.24 | -0.84 – 0.11 | -1.51 | .132 |
| Group (BPD) | -0.23 | 0.25 | -0.72 – 0.27 | -0.90 | .369 |
| Outcome (Loss) | 0.01 | 0.06 | -0.11 – 0.13 | 0.13 | .894 |
| Gender (Women) | 0.10 | 0.25 | -0.39 – 0.59 | 0.41 | .683 |
| Trial z | -0.04 | 0.03 | -0.10 – 0.03 | -1.19 | .233 |
| Group (MDD) × Outcome (Loss) | 0.13 | 0.08 | -0.03 – 0.29 | 1.62 | .105 |
| Group 3(BPD) × Outcome (Loss) | 0.09 | 0.08 | -0.07 – 0.26 | 1.09 | .276 |
| **Model 2 (SCR)**  *rlmer (SCR ~ Aggrchoice + Group + Outcome + Gender + Trial.z + Group*Aggrchoice +*  *Group*Outcome+ (1+Trial.z\|Subject))* | | | | | |
| **Fixed effects:** | | | | | |
| Intercept | 0.24 | 0.05 | 0.15 – 0.33 | 5.24 | <.001*** |
| Aggrchoice | -0.01 | 0.01 | -0.03 – 0.01 | -1.04 | .298 |
| Group (MDD) | -0.01 | 0.04 | -0.09 – 0.08 | -0.12 | .906 |
| Group (BPD) | -0.03 | 0.05 | -0.12 – 0.06 | -0.67 | .504 |
| Outcome (Loss) | 0.01 | 0.02 | -0.02 – 0.04 | 0.35 | .729 |
| Gender (Women) | -0.03 | 0.03 | -0.09 – 0.04 | -0.79 | .432 |
| Trial z | -0.06 | 0.01 | -0.07 – -0.04 | -6.71 | <.001*** |
| Aggrchoice × Group (MDD) | 0.00 | 0.01 | -0.02 – 0.02 | 0.10 | .921 |
| Aggrchoice × Group (BPD) | 0.02 | 0.01 | 0.00 – 0.05 | 2.08 | .038* |
| Group (MDD) × Outcome (Loss) | -0.01 | 0.02 | -0.05 – 0.03 | -0.71 | .478 |
| Group (BPD) × Outcome (Loss) | -0.01 | 0.02 | -0.05 – 0.03 | -0.58 | 0.559 |

Note. *b*: estimate; *SE*, Standard Error; *CI*, Confidence Intervals; Aggrchoice, state proactive aggression; Outcome, game outcome in the previous trial; HC, healthy controls; MDD, major depressive disorder, BPD: borderline personality disorder; Trial.z: z-transformed trials.* *p* < .05; *** *p* < .001.

**Supplementary Table 4.** Comparisons between the two transdiagnostic groups, LPA (low proactive aggression) and HPA (high proactive aggression).

|  | **LPA (*n* = 36)** | **HA (*n* = 28)** | ***Stats.*** | ***p*** |
| --- | --- | --- | --- | --- |
| HC | 15 | 6 |  | > .05 |
| MDD | 11 | 12 |  | > .05 |
| BPD | 10 | 10 |  | > .05 |
| Gender (women) | 29 | 22 | *X*^2^ (1, *N* = 64) = 0.04 | .85 |
| Belief in cover story (yes)^a^ | 18 | 12 | *X*^2^ (1, *N* = 62) = 0.63 | .43 |
| State proactive aggression (pTAP, choices from 1 to 4) | 2.22 ± 0.72 | 2.21 ± 0.74 | *t* (62) = 0.06 | .96 |
| BDI-II | 17.75 ± 15.97 | 24.79 ± 13.94 | *U* = 356.5 | < .05* |
| RPQre | 5.92 ± 4.25 | 9.04 ± 4.48 | *t* (62) = -2.845 | < .01** |
| Hostility (BPAQ) | 16.42 ± 6.73 | 18.57 ± 6.22 | *U* = 411 | .21 |
| Anger (BPAQ) | 15.17 ± 4.80 | 17.64 ± 5.63 | *U* = 379.5 | .09 |
| Physical aggression (BPAQ) | 15.19 ± 4.48 | 16.54 ± 4.96 | *U* = 415 | .23 |
| Verbal aggression (BPAQ) | 11.25 ± 3.60 | 13.46 ± 3.84 | *U* = 319.5 | < .05* |
| SCR (decision phase)^b^ | 0.28 ± 0.24 | 0.23 ± 0.22 | *U* = 402 | .50 |

pTAP, proactive Taylor Aggression Paradigm; SCR, skin conductance response.

^a^ Data regarding Belief in cover story were missing for 2 participants in the LPA group.

^b^ Data regarding SCR were missing for 4 participants in the LPA group

**Supplementary Table 5.**Parameter estimates from the linear mixed-effects model analyses for aggression choices (Model S1) and for SCR (Model S2) in women.

| **Model S1 (Aggrchoice)**  *rlmer (Aggrchoice ~ Group + Outcome + Trial.z + Group*Outcome + (1 + Trial.z\|Subject))* | | | | | |
| --- | --- | --- | --- | --- | --- |
| **Fixed effects:** | ***b*** | ***SE*** | ***95% CI*** | ***t*** | ***p*** |
| Intercept | 2.37 | 0.18 | 2.03 – 2.71 | 13.51 | <.001*** |
| Group (MDD) | -0.46 | 0.25 | -0.94 – 0.02 | -1.86 | .063 |
| Group (BPD) | -0.14 | 0.25 | -0.62 – 0.35 | -0.55 | .581 |
| Outcome (Loss) | -0.04 | 0.07 | -0.18 – 0.10 | -0.58 | .562 |
| Trial z | -0.04 | 0.04 | -0.12 – 0.03 | -1.08 | .279 |
| Group (MDD) × Outcome (Loss) | 0.29 | 0.10 | 0.10 – 0.49 | 2.92 | **.003**** |
| Group 3(BPD) × Outcome (Loss) | 0.14 | 0.10 | -0.06 – 0.34 | 1.40 | .161 |
| **Model S2 (SCR)**  *rlmer (SCR ~ Aggrchoice + Group + Outcome + Trial.z + Group*Aggrchoice + Group*Outcome +*  *(1+Trial.z\|Subject))* | | | | | |
| **Fixed effects:** | | | | | |
| Intercept | 0.22 | 0.04 | 0.14 – 0.30 | 5.34 | **<.001***** |
| Aggrchoice | -0.01 | 0.01 | -0.03 – 0.01 | -1.00 | .318 |
| Group (MDD) | -0.02 | 0.05 | -0.12 – 0.07 | -0.51 | .611 |
| Group (BPD) | -0.01 | 0.05 | -0.10 – 0.09 | -0.12 | .904 |
| Outcome (Loss) | 0.01 | 0.02 | -0.03 – 0.04 | 0.34 | .732 |
| Trial z | -0.07 | 0.01 | -0.09 – -0.04 | -5.68 | <.001*** |
| Aggrchoice × Group (MDD) | 0.01 | 0.01 | -0.02 – 0.03 | 0.46 | .647 |
| Aggrchoice × Group (BPD | 0.02 | 0.01 | -0.00 – 0.04 | 1.59 | .111 |
| Group (MDD) × Outcome (Loss) | -0.02 | 0.02 | -0.06 – 0.03 | -0.78 | .434 |
| Group (BPD) × Outcome (Loss) | -0.01 | 0.02 | -0.06 – 0.03 | -0.65 | .518 |

Note. *b*: estimate; *SE*, Standard Error; *CI*, Confidence Intervals; Aggrchoice, state proactive aggression; Outcome, game outcome in the previous trial; HC, healthy controls; MDD, major depressive disorder, BPD: borderline personality disorder; Trial.z: z-transformed trials. ** *p* < .01; *** *p* < .001.

**Supplementary Table 6**. Post hoc test on the interaction between *Group* and *Outcome* in model S1 (aggression choices) in the women group.

|  | **Estimate** | **SE** | ***z*** | ***p*** |
| --- | --- | --- | --- | --- |
| *Outcome = Won* |  |  |  |  |
| HC - MDD | 0.459 | 0.247 | 1.861 | .150 |
| HC - BPD | 0.136 | 0.246 | 0.552 | .846 |
| MDD - BPD | -0.323 | 0.246 | -1.312 | .388 |
| *Outcome = Loss*  HC - MDD | 0.165 | 0.247 | 0.670 | .781 |
| HC - BPD | -0.006 | 0.247 | -0.023 | 1.00 |
| MDD - BPD | -0.171 | 0.247 | -0.693 | .767 |

*Note.* SE, Standard Error.

## Supplementary Figures

**
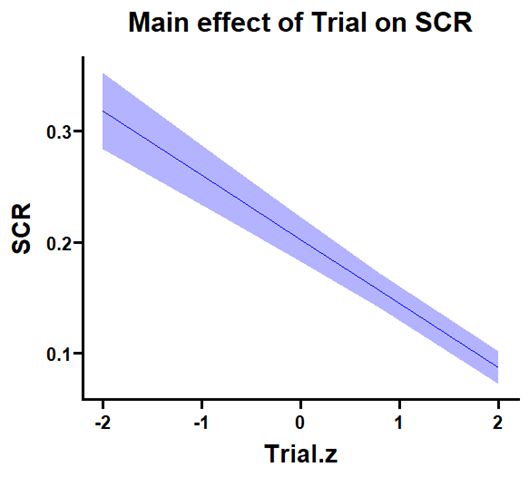
**

**Supplementary Figure 1.** Main effect *Trial.z* in the SCR model (Model 2). Trial.z: z-transformed trials. SCR: skin conductance response.


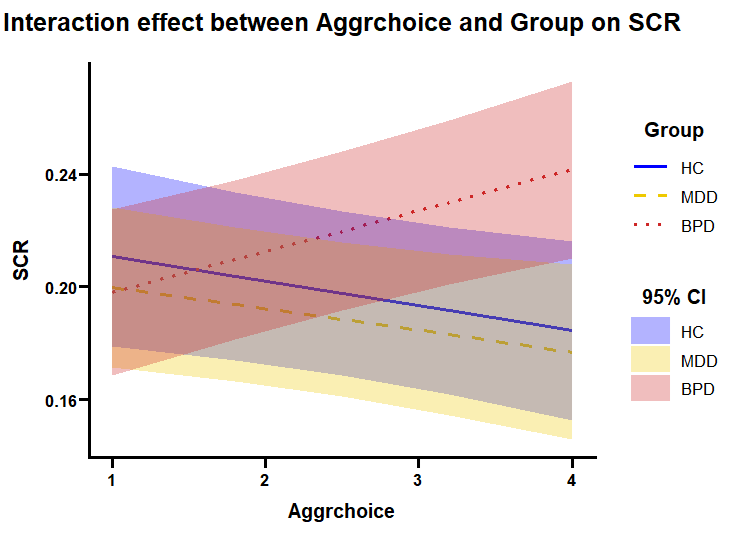


**Supplementary Figure 2.** Interaction effect between Aggrchoice and Group in the SCR model (Model 2). SCR: skin conductance response, HC: healthy controls, MDD: major depressive disorder; BPD: borderline personality disorder. The post-hoc tests were not significant.


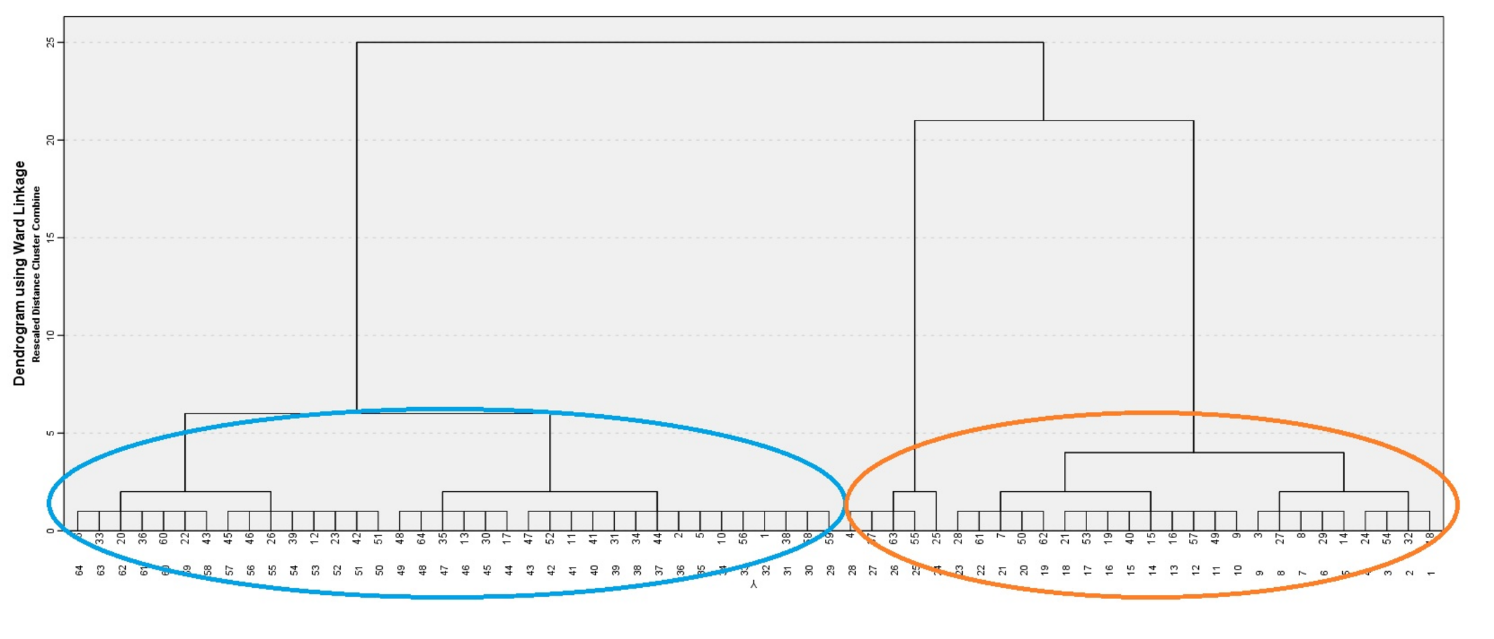


**Supplementary Figure 3.** Dendrogram showing the two identified transdiagnostic groups: blue: Low Aggression group (LPA); orange: High Aggression group (HPA).
